# Supplementary figures and images for: Nuclear fascin regulates cancer cell survival
Source: eLife. 2022 Aug 30;11:e79283. doi: 10.7554/eLife.79283 (PMC9427113; doi:10.7554/eLife.79283)

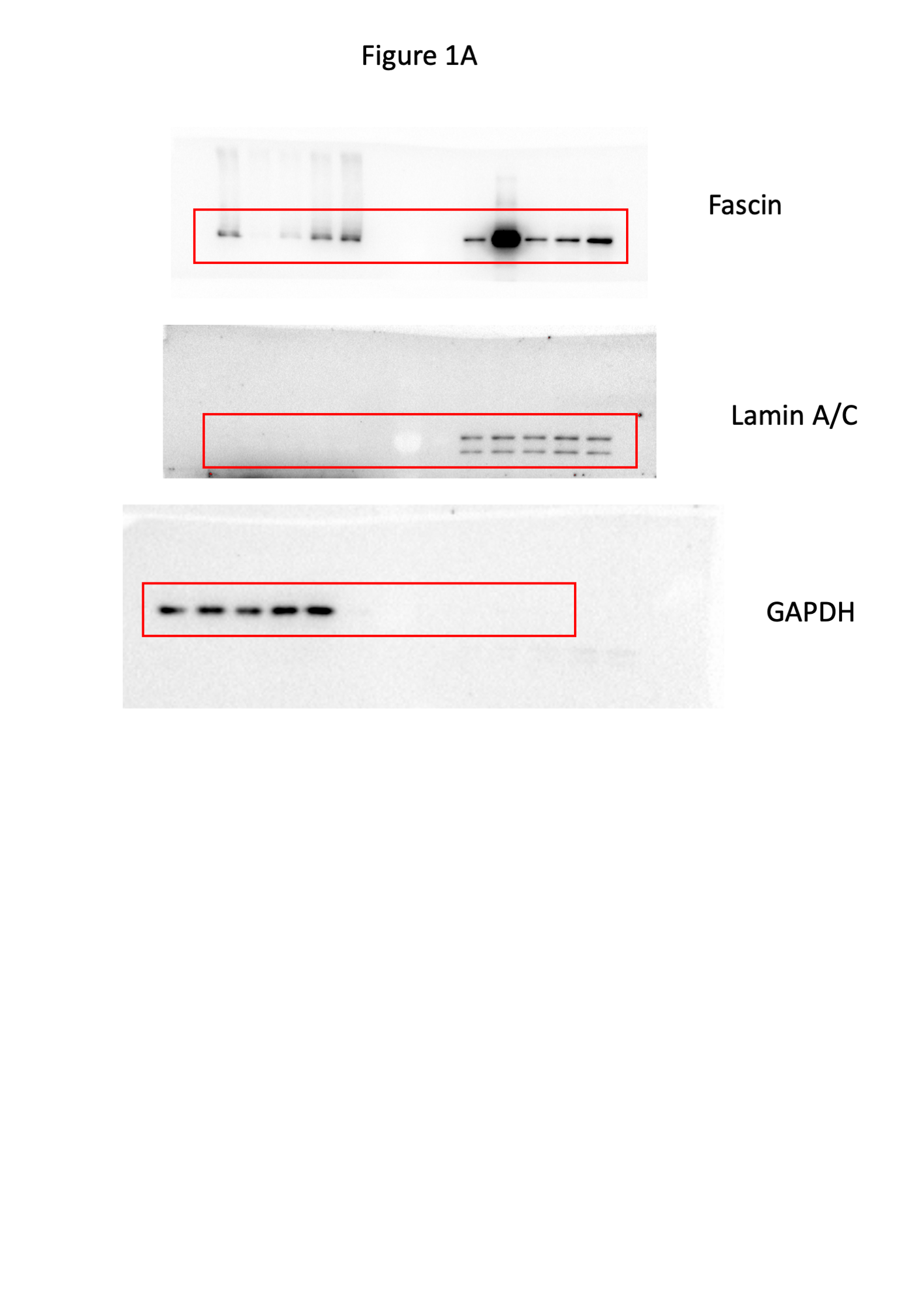

Supplement: Figure 1—source data 1. [file elife-79283-fig1-data1.tiff]

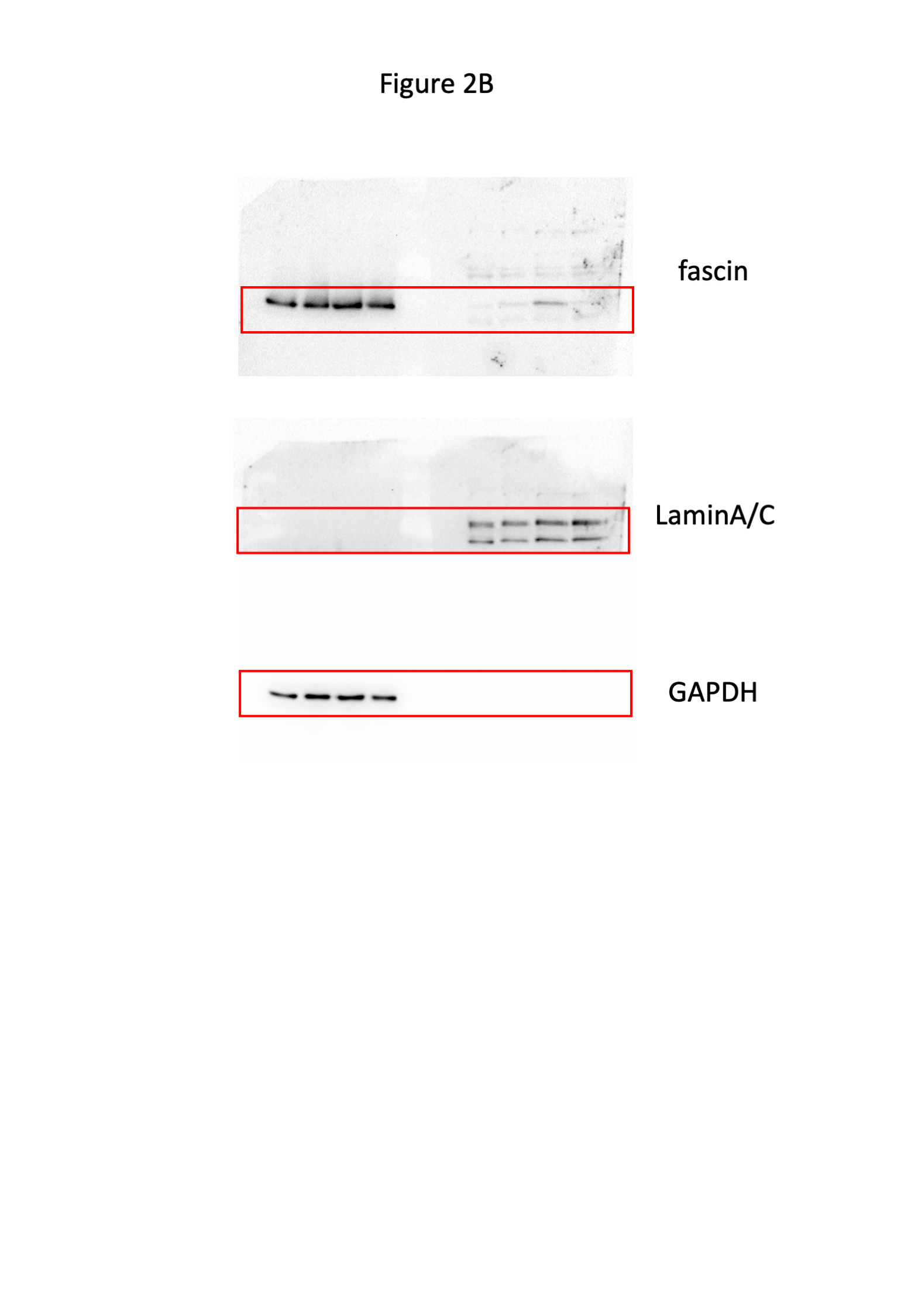

Supplement: Figure 2—source data 1. [file elife-79283-fig2-data1.tiff]

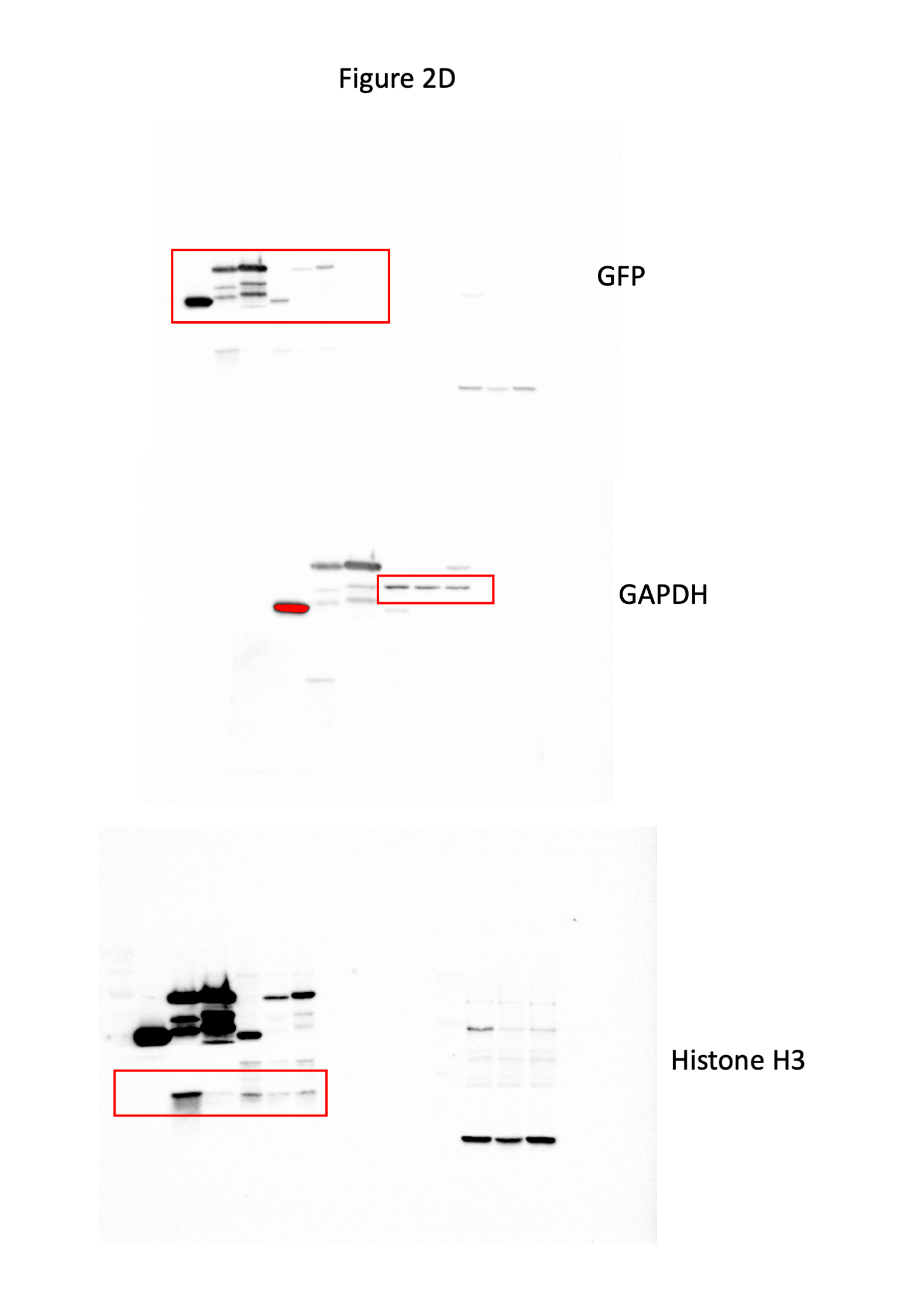

Supplement: Figure 2—source data 3. [file elife-79283-fig2-data3.tiff]

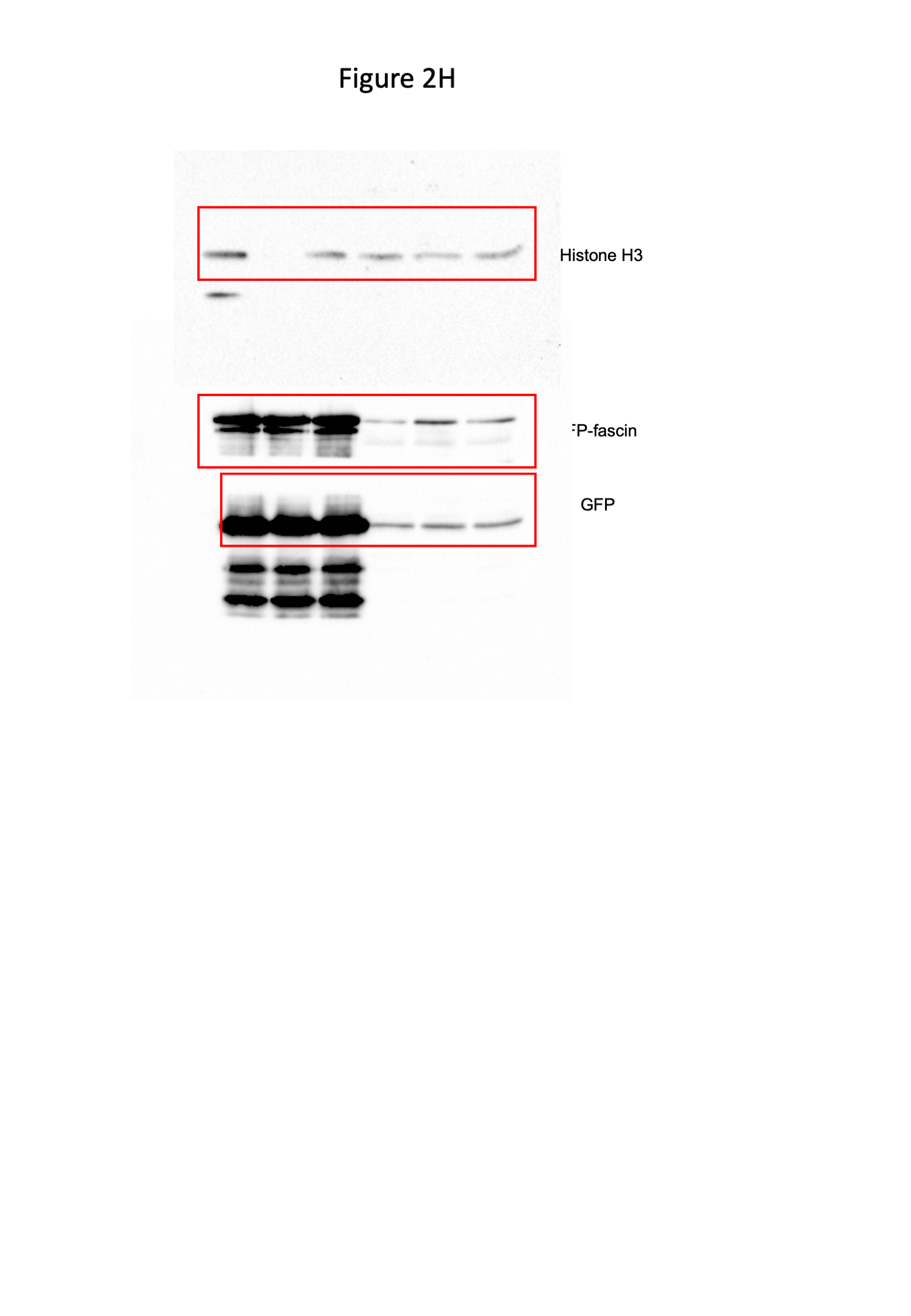

Supplement: Figure 2—source data 4. [file elife-79283-fig2-data4.tiff]

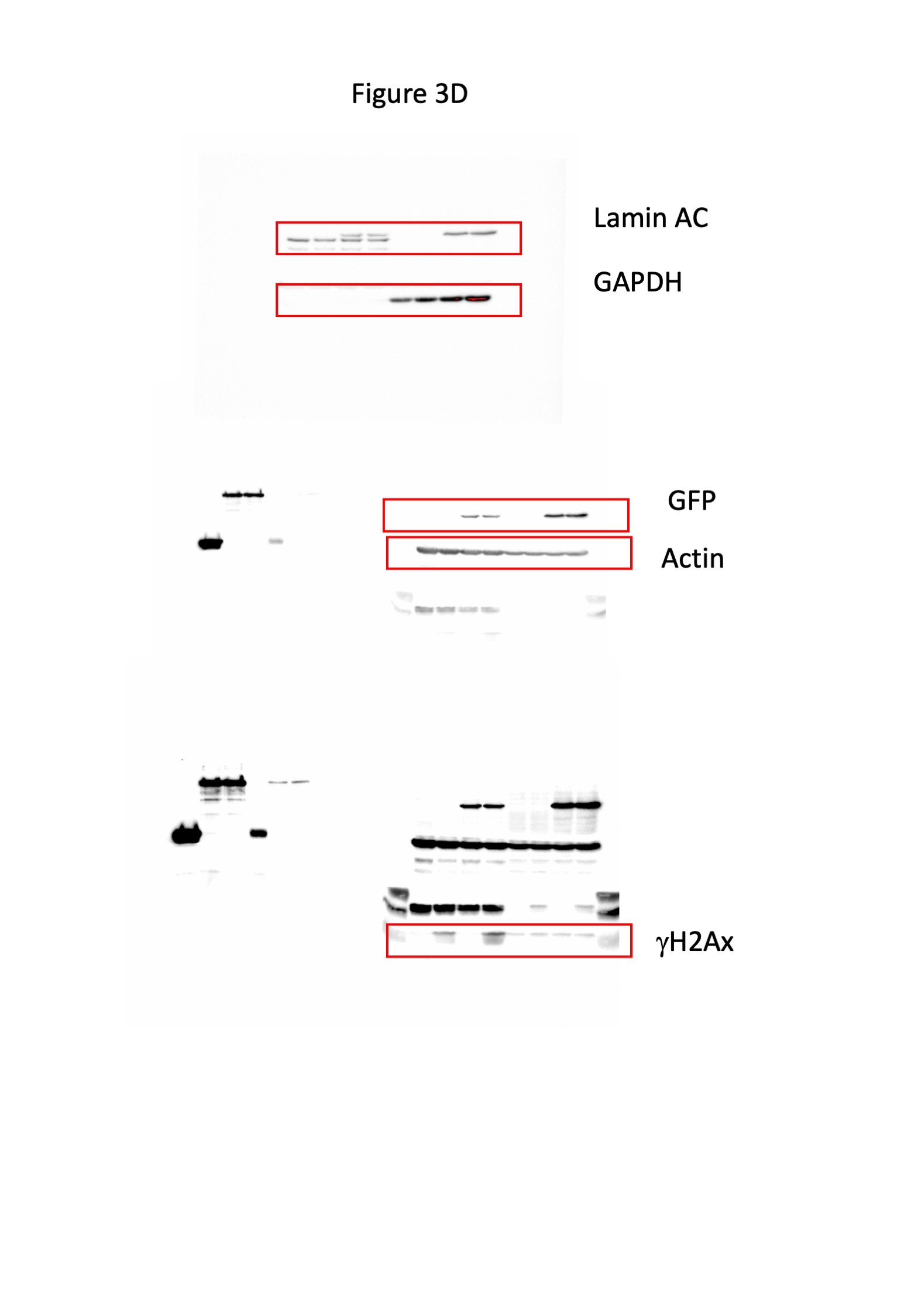

Supplement: Figure 3—source data 1. [file elife-79283-fig3-data1.tiff]

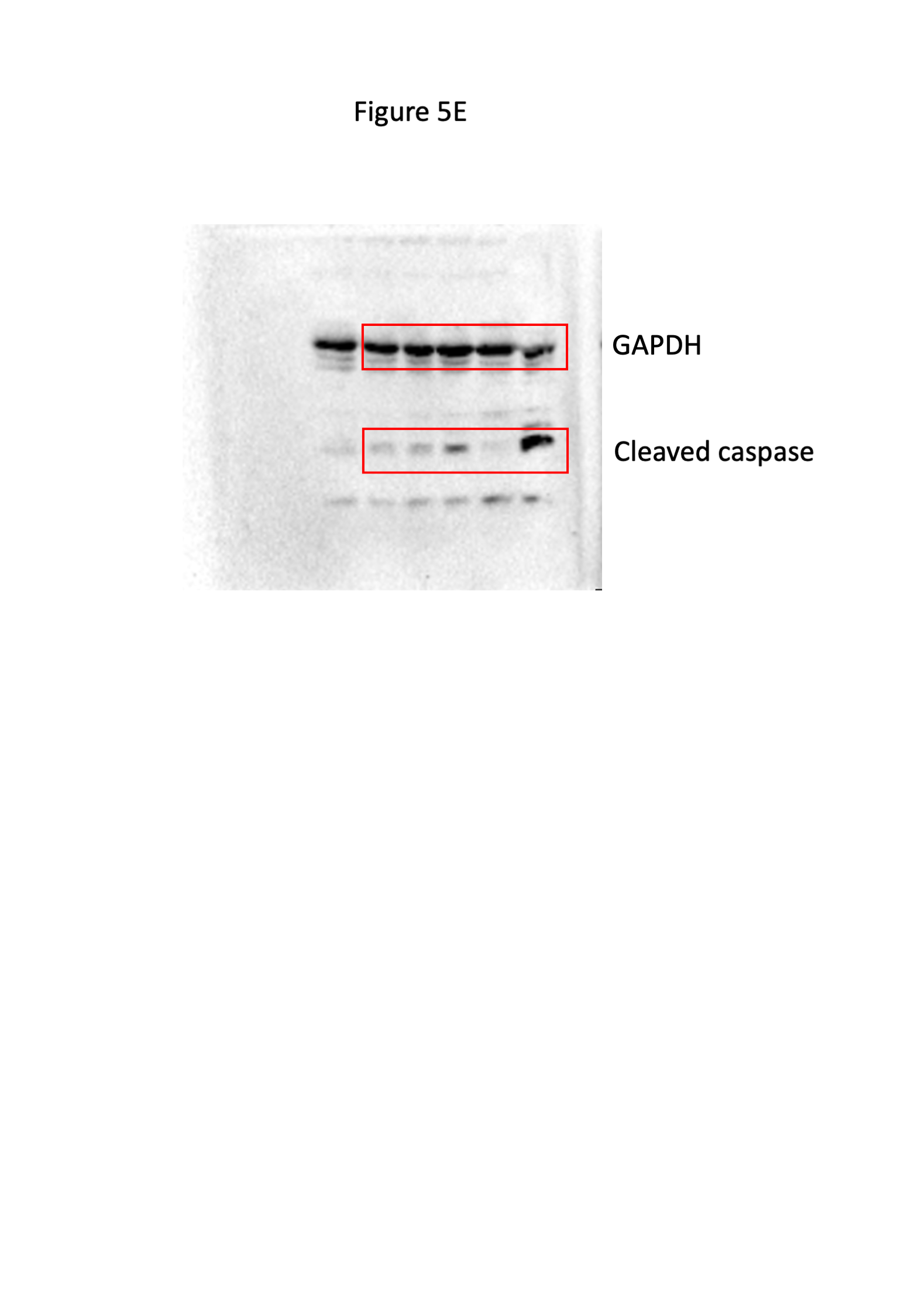

Supplement: Figure 5—source data 1. [file elife-79283-fig5-data1.tiff]

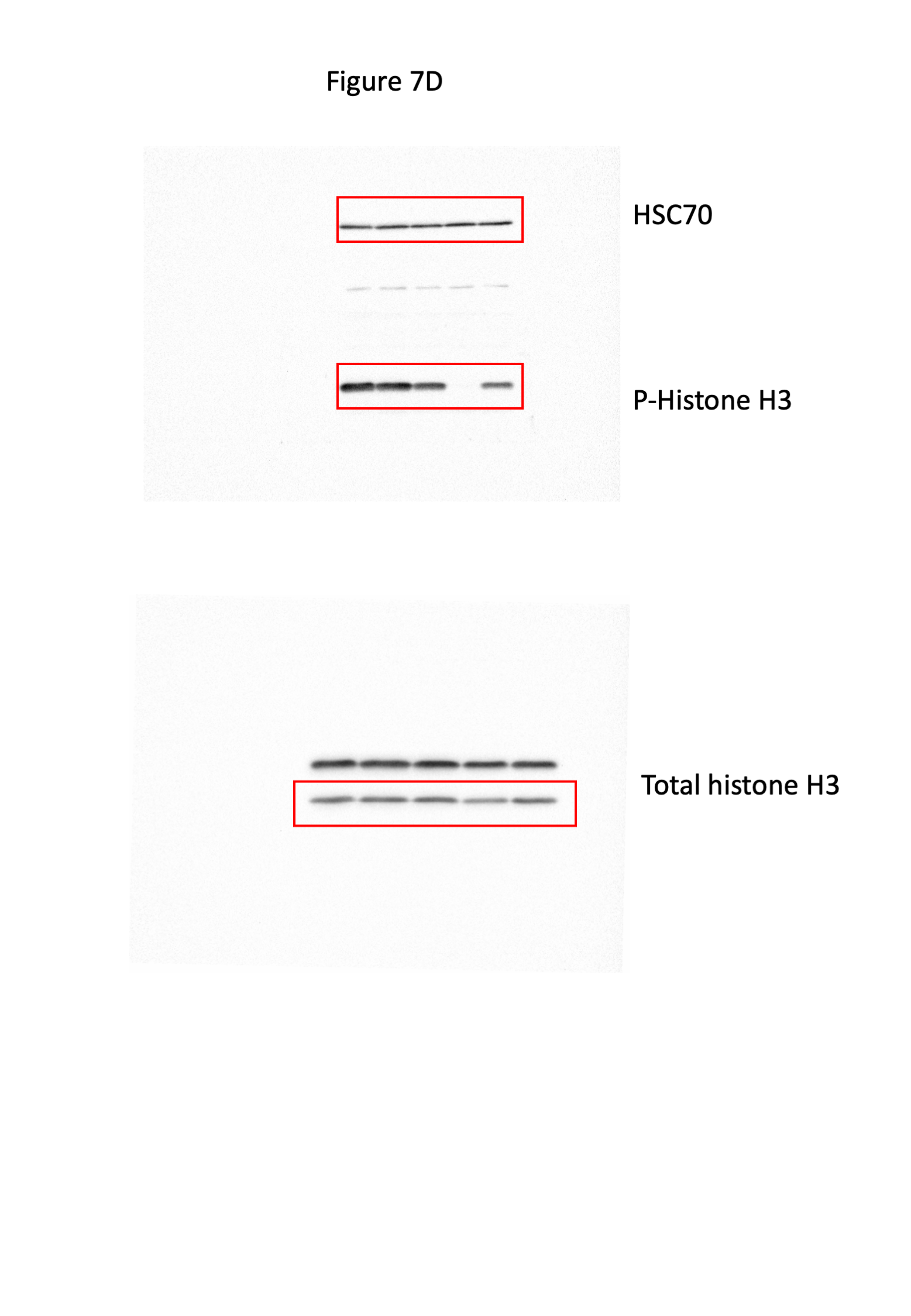

Supplement: Figure 7—source data 3. [file elife-79283-fig7-data3.tiff]

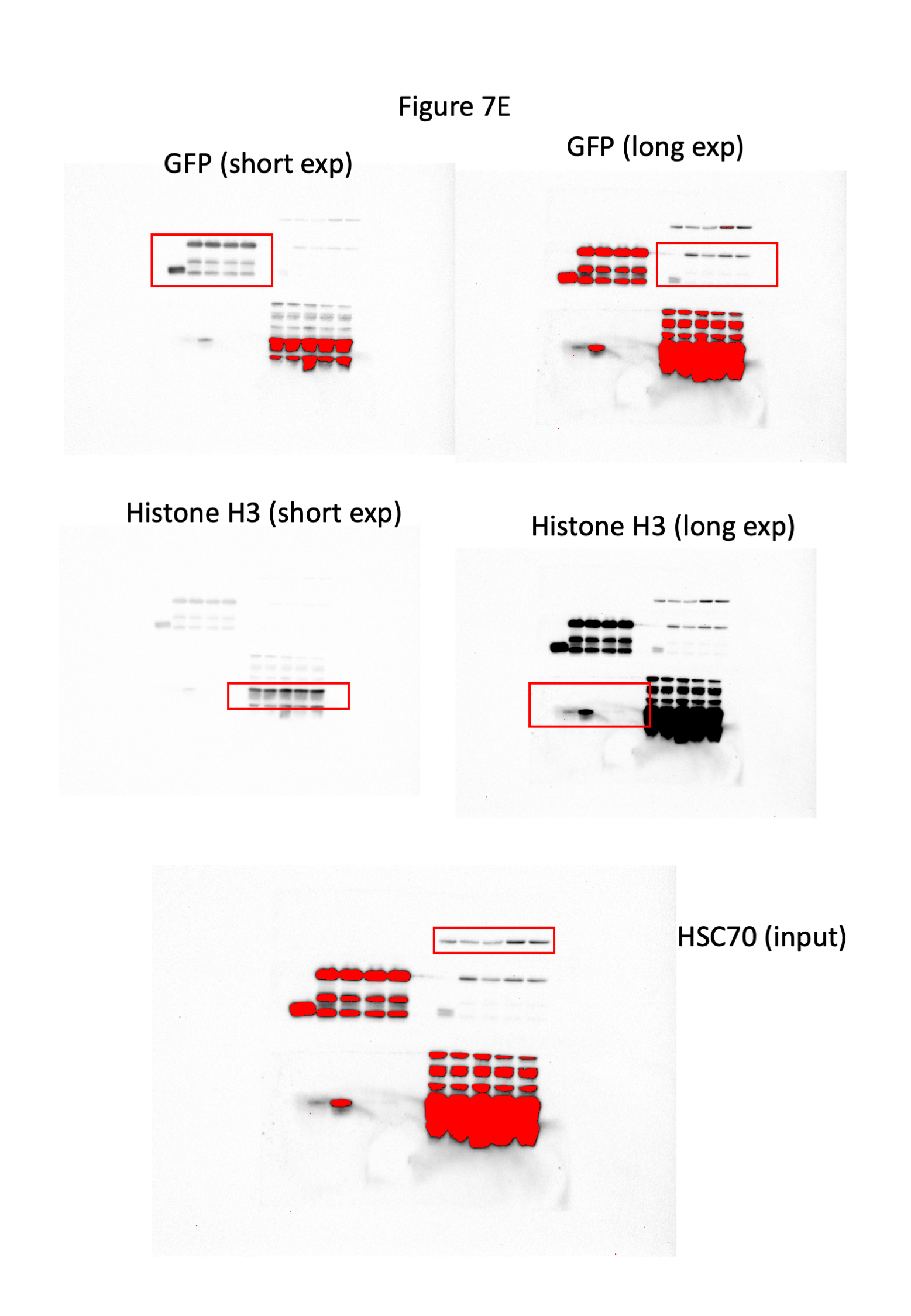

Supplement: Figure 7—source data 4. [file elife-79283-fig7-data4.tiff]

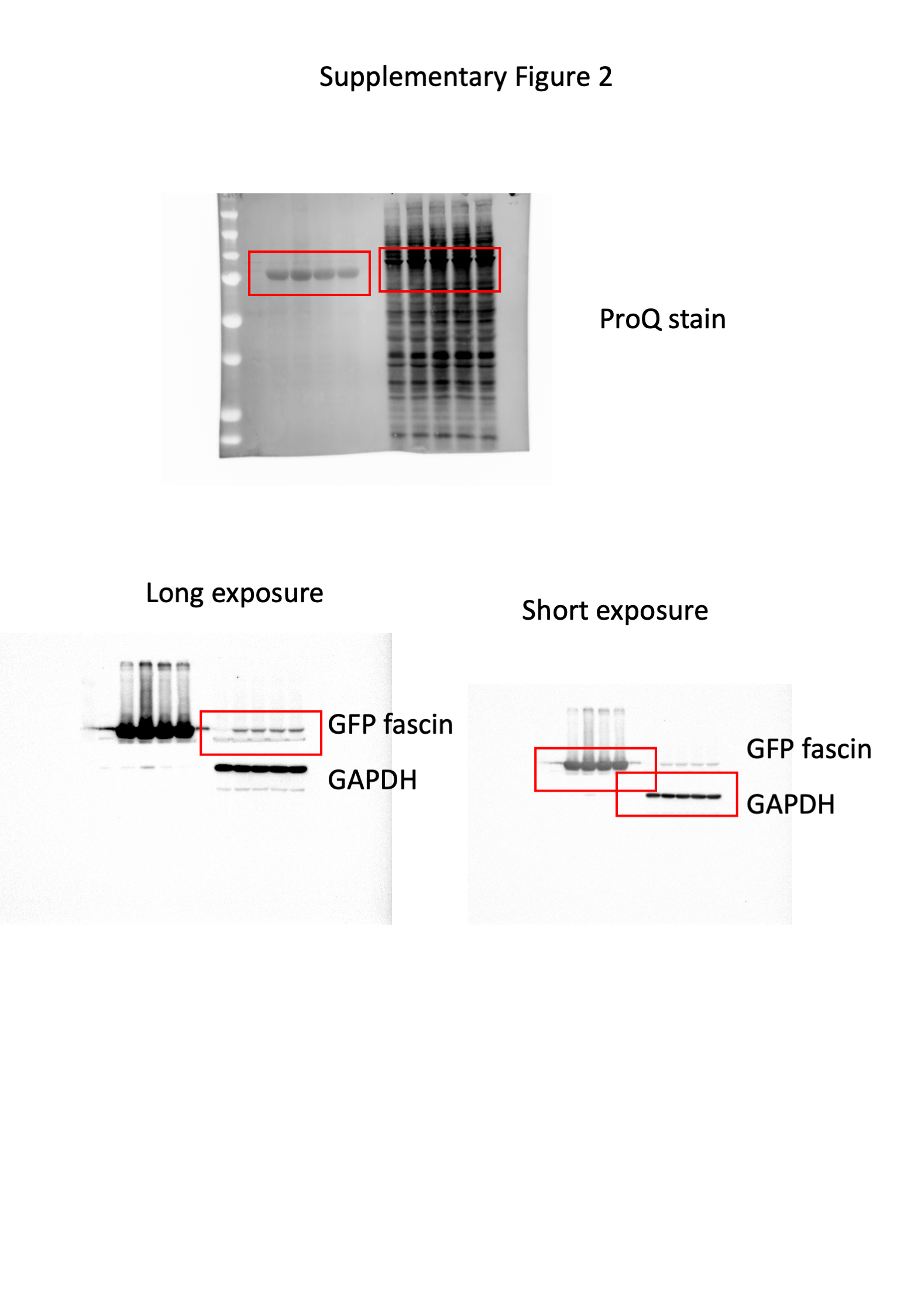

Supplement: Figure 7—figure supplement 1—source data 1. [file elife-79283-fig7-figsupp1-data1.tiff]
